# Supplementary material for: FERN – a Java framework for stochastic simulation and evaluation of reaction networks
Source: BMC Bioinformatics. 2008 Aug 29;9:356. doi: 10.1186/1471-2105-9-356 (PMC2553347; doi:10.1186/1471-2105-9-356)
Supplement: Additional file 1 — FERN distribution, Version 1.3. This archive contains the FERN source code and binaries as well as documentation and example models in FernML and SBML. [file 1471-2105-9-356-S1.zip › fern/doc/javadoc/fern/simulation/algorithm/AbstractTauLeapingPropensityBoundSimulator.html]

AbstractTauLeapingPropensityBoundSimulator


---


|  |  |  |
| --- | --- | --- |
| **Overview** \| **Package** \| **Class** \| **Use** \| **Tree** \| **Deprecated** \| **Index** \| **Help** | |  |
| **PREV CLASS**   **NEXT CLASS** | **FRAMES**    **NO FRAMES**     **All Classes** |  |
| SUMMARY: NESTED \| FIELD \| CONSTR \| METHOD | DETAIL: FIELD \| CONSTR \| METHOD |  |


---


## fern.simulation.algorithm Class AbstractTauLeapingPropensityBoundSimulator

```
java.lang.Object
  fern.simulation.Simulator
      fern.simulation.algorithm.GillespieEnhanced
          fern.simulation.algorithm.AbstractBaseTauLeaping
              fern.simulation.algorithm.AbstractTauLeapingPropensityBoundSimulator
```

**Direct Known Subclasses:**: TauLeapingAbsoluteBoundSimulator, TauLeapingRelativeBoundSimulator

---

``` public abstract class AbstractTauLeapingPropensityBoundSimulator extends AbstractBaseTauLeaping ```

There are some possibilities to bind the expected change of the propensities by a
value epsilon in order to fulfill the leap condition. This base class provides
mathematics for binding it directly by examining the actual propensities.

Daniel T. Gillespie, Approximate accelerated stochastic simulation
of chemically reacting systems, Journal of chemical physics vol 115, nr 4 (2001); Cao et al., Efficient
step size selection for the tau-leaping simulation method, Journal of chemical physics 124, 044109 (2006)

**Author:**
:   Florian Erhard

---

**Nested Class Summary**

**Nested classes/interfaces inherited from class fern.simulation.Simulator**

`Simulator.FireType`


| **Field Summary** | |
| --- | --- |
| `protected  double[][]` | `f` |
| `protected  double[]` | `mu` |
| `protected  double[]` | `sigma` |

**Fields inherited from class fern.simulation.algorithm.AbstractBaseTauLeaping**

`productHistos, reactantHistos, verbose`

**Fields inherited from class fern.simulation.algorithm.GillespieEnhanced**

`a_sum`

**Fields inherited from class fern.simulation.Simulator**

`a, stochastics, t`


**Constructor Summary**

`AbstractTauLeapingPropensityBoundSimulator(Network net)`


| **Method Summary** | |
| --- | --- |
| `protected  double` | `chooseTauNonCriticals(BitVector criticals)`             choose the timestep candidate for the noncritical reactions by some method. |
| `protected abstract  double` | `getTop(int j)` |

**Methods inherited from class fern.simulation.algorithm.AbstractBaseTauLeaping**

`chooseTauCriticals, getEpsilon, getLangevinThreshold, getName, getNCritical, getNumSimpleCalls, getUseSimpleFactor, getV, isVerbose, performStep, setEpsilon, setLangevinThreshold, setNCritical, setNumSimpleCalls, setUseSimpleFactor, setVerbose`

**Methods inherited from class fern.simulation.algorithm.GillespieEnhanced**

`directMCTau, initialize, isEfficientlyAdaptSum, reinitialize, setAmount, setEfficientlyAdaptSum, setVolume`

**Methods inherited from class fern.simulation.Simulator**

`addObserver, fireReaction, fireReaction, getAmount, getAmountManager, getNet, getNextThetaEvent, getPropensity, getPropensityCalculator, getTime, getVolume, isInterpolateTheta, registerNewTheta, setInterpolateTheta, start, start, thetaEvent`

**Methods inherited from class java.lang.Object**

`clone, equals, finalize, getClass, hashCode, notify, notifyAll, toString, wait, wait, wait`

**Field Detail**

### f

```
protected double[][] f
```

---


### mu

```
protected double[] mu
```

---


### sigma

```
protected double[] sigma
```


**Constructor Detail**

### AbstractTauLeapingPropensityBoundSimulator

```
public AbstractTauLeapingPropensityBoundSimulator(Network net)
```


**Method Detail**

### chooseTauNonCriticals

```
protected double chooseTauNonCriticals(BitVector criticals)
```

:   **Description copied from class: `AbstractBaseTauLeaping`**
:   choose the timestep candidate for the noncritical reactions by some method.

    :   **Specified by:**: `chooseTauNonCriticals` in class `AbstractBaseTauLeaping`
    :   **Parameters:**: `criticals` - Bitvector identifying the critical reactions **Returns:**: timestep candidate for noncritical reactions

---


### getTop

```
protected abstract double getTop(int j)
```


---


|  |  |  |
| --- | --- | --- |
| **Overview** \| **Package** \| **Class** \| **Use** \| **Tree** \| **Deprecated** \| **Index** \| **Help** | |  |
| **PREV CLASS**   **NEXT CLASS** | **FRAMES**    **NO FRAMES**     **All Classes** |  |
| SUMMARY: NESTED \| FIELD \| CONSTR \| METHOD | DETAIL: FIELD \| CONSTR \| METHOD |  |


---
